# Volume expansion of periaqueductal gray in episodic migraine: a pilot MRI structural imaging study

**DOI:** 10.1186/s10194-017-0797-z

**Published:** 2017-08-15

**Authors:** Zhiye Chen, Xiaoyan Chen, Mengqi Liu, Shuangfeng Liu, Lin Ma, Shengyuan Yu

**Affiliations:** 1 Department of Radiology, Chinese PLA General Hospital, Fuxing Road 28, Beijing, 100853 China; 2 Department of Neurology, Chinese PLA General Hospital, Fuxing Road 28, Beijing, 100853 China; 3 Department of Radiology, Hainan Branch of Chinese PLA General Hospital, Sanya, 572013 China

**Keywords:** Chronic migraine, Episodic migraine, Periaqueductal gray, Magnetic resonance imaging, Volume measurement

## Abstract

**Background:**

The periaqueductal gray (PAG) dysfunction was recognized in migraine, and the nonspecific PAG lesions were also observed in episodic migraine (EM) recently. However, the PAG volume change was not totally detected in EM up to now. Herein, the aim of this study was to investigate altered PAG volume in EM patients based on high resolution brain structural image.

**Methods:**

The brain structural images were obtained from 18 normal controls (NC), 18 EM patients and 16 chronic migraine (CM) on 3.0 T MR system. PAG template was created based on the ICBM152 gray matter template using MRIcron, and the individual PAG was created by applying the deformation field to the PAG template after structural image segment. One-way analysis of covariance, partial correlation analysis and Receiver operating characteristics (ROC) curve were applied.

**Results:**

EM had a larger PAG volume (0.35 ± 0.02 ml) than that (0.32 ± 0.02 ml) of NC (*P* = 0.017). The PAG volume of CM (0.33 ± 0.02 ml) was negatively related to the VAS score (*P* = 0.03). ROC analysis demonstrated that PAG volume has higher diagnostic efficacy (AUC, 0.731; Sensitivity, 0.556; Specificity, 0.889) for NC vs. EM compared with that NC vs. CM (AUC, 0.634; Sensitivity, 0.438; Specificity, 0.833) and EM vs. CM (AUC, 0.618; Sensitivity, 0.813; Specificity, 0.556).

**Conclusion:**

PAG volume expansion may be the direct impairment evidence on the brain in EM, and could be considered as a diagnostic and evaluated imaging biomarker in migraine.

## Background

The Migraine is a common type of primary headaches with a reported prevalence of approximately 5.7% in men and 17.0% in women [1], and affect 12% of the population worldwide [2]. The neuromechanism of migraine has been the key focus of research [3]. Of all the target “generator” of migraine attacks, the PAG region has been the key observed brain structure.

Periaqueductal gray (PAG) was a center with powerful descending antinociceptive neuronal network in midbrain [4, 5], and PAG activation was modulated by expectation of pain [6] and placebo analgesia [7]. PAG could exert a dual control, including inhibition and facilitation, on nociceptive transmission in the dorsal horn and trigeminal nucleus [8] by descending PAG-RVM (rostral ventromedial medulla) pathway contributing to central sensitization and development of secondary hyperalgesia [8, 9]. A previous study [10] confirmed PAG dysfunction in migraine, and functional MRI studies demonstrated that the PAG dysfunction was associated with increased iron deposition, which may play a role in the genesis or pathophysiology of MOH [4, 11, 12] The PAG dysfunction changes might explain the neuromechanism of migraine, however, the PAG structure change was not elucidated completely.

PAG abnormalities can be detected in migraine patients with brain T2-visible lesion using voxel-based morphometry (VBM), which mainly identified increased PAG density in migraine brain [13]. The altered PAG density indicated the volume change without modulation in VBM, which did not represent the true volume change [14]. Therefore, the true PAG volume abnormalities were not investigated in episodic migraine.

Although PAG was a very small region in the midbrain, and the PAG volume changes had indirectly been assessed by VBM [13, 15, 16], which represent the volume changes in statistical level while not the true volume changes. Therefore, the PAG volume measurement was important for the accurate structural assessment of PAG. In our previous study, the PAG volume measurement using automated PAG segment had been applied to the medication-overuse patients [17].

In the current study, we hypothesize that migraine patients without T2-visible lesions may present PAG volume changes. To address this hypothesis, we prospectively obtained conventional T2WI and high resolution structural images from 18 episodic migraine (EM) patients, 16 chronic migraine (CM) patients and 18 age- and sex-matched normal controls without T2-visible lesions on the brain to calculate and analyze PAG volume change using an automated three dimensional volume mapping measurement.

## Methods

### Subjects

Written informed consent was obtained from all participants according to the approval of the ethics committee of the local institutional review board. Eighteen EM patients without aura and 16 chronic migraine (CM) patients without aura were recruited from the International Headache Center, Department of Neurology, Chinese PLA General Hospital. The diagnostic criteria of EM and CM should meet the following conditions: (1) EM is defined as migraine attack days being less than 15 days per month [18]. The definition of migraine refers to 1.1 Migraine without aura and 1.2 Migraine with aura in ICHD-III beta [19]; (2) diagnosis of 1.3 CM, and 1.1 and 1.2 migraine based on the International Classification of Headache Disorders, third Edition (beta version) (ICHD-III beta) [19]; (3) no migraine preventive medication used in the past 3 months; The patients should be excluded if they meet the following conditions: (1) with any chronic disorders such as hypertension, diabetes mellitus and cerebrovascular disease; (2) with alcohol, nicotine, or other substance abuse; (3) with any cerebral disorder.

Eighteen NCs were recruited from the hospital’s staff and their relatives. NCs should never have any primary headache disorders or other types of headache in the past year, and fulfil the same exclusion criteria. Additionally, the anxiety, depression, and cognitive function of all the participants were assessed by using the Hamilton Anxiety Scale (HAMA) [20], the Hamilton Depression Scale (HAMD) [21], and the Montreal Cognitive Assessment (MoCA) Beijing Version (www.mocatest.org). MRI scans were taken in the interictal stage at least three days after a migraine attack for EM patients. All the patients were given with the Visual Analogue Scale (VAS) and the Migraine Disability Assessment Scale (MIDAS). All the subjects underwent conventional MRI examination to exclude the subjects with cerebral infarction, malacia, or occupying lesions. Alcohol, nicotine, caffeine, and other substances were avoided for at least 12 h before MRI examination.

### MRI acquisition

Images were acquired on a GE 3.0 T MR system and a conventional eight-channel quadrature head coil was used. All subjects were instructed to lie in a supine position, and formed padding was used to limit head movement. The structural images were generated by a three-dimensional T1-weighted fast spoiled gradient recalled echo (3D T1-FSPGR) sequence, and the scanning parameters were set as follows: TR (repetition time) = 6.3 ms, TE (echo time) = 2.8 ms, flip angle = 15o, FOV (field of view) = 25.6 cm × 25.6 cm, Matrix = 256 × 256, NEX (number of acquisition) = 1. All imaging protocols were identical for all subjects.

### MR image processing

**Fig. 1:**
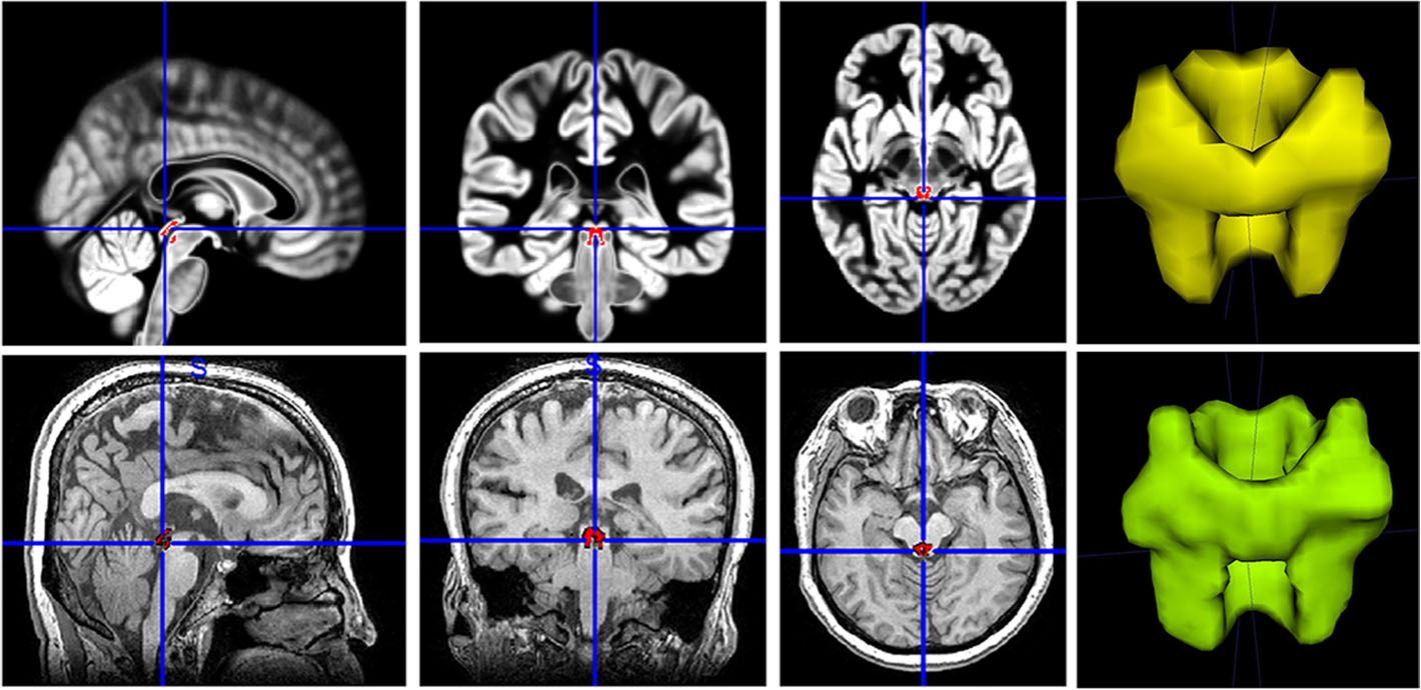
The creation of PAG template and individual PAG. Top line represents the PAG template created by MRIcron based on mni_icbm152_gm_tal_nlin_asym_09a template. Bottom line represents the individual PAG created by deformation field. The last column represent three-dimensional reconstructed image of PAG template and individual PAG, which were created by ITK-SNAP (version 3.6.0 beta) (http://www.itksnap.org)

All MR structural image data were processed using Statistical Parametric Mapping 12 (SPM 12) (http://www.fil.ion.ucl.ac.uk/spm/) running under MATLAB 7.6 (The Mathworks, Natick, MA, USA). The image processing included following steps: (1) Create PAG template based on mni_icbm152_gm_tal_nlin_asym_09a template using MRIcron; (2) Create individual PAG mask by apply the deformation field (generated by new segment) to the PAG template using run-back strategy; (3) compute the PAG volume by ITK-SNAP (version 3.6.0 beta) (http://www.itksnap.org) (Fig. 1).

### Statistical analysis

The statistical analysis was performed by using PASW Statistics 18.0. One-way analysis of covariance was performed among each group with age as covariate. Partial correlation were performed between the PAG volume and the clinical variables with age as covariate. Significant difference was set at a *P* value of <0.05. Receiver operating characteristics (ROC) curve was applied to evaluate the diagnostic efficacy of PAG volume, and area under the curve (AUC) was recognized reasonable diagnostic valuable with AUC set at >0.7.

## Results

### Demography and neuropsychological test

Demographic and clinical data are summarized in Table 1. Eighteen EM patients (F/M = 14/4), 16 CM patients (F/M = 14/2) and 18 NCs (F/M = 14/4) were enrolled. There was a significant difference for age between EM (33.39 ± 10.69 years old) and CM (42.44 ± 8.65 years old). There was a significant difference for HAMA between NC (9.67 ± 3.16) and EM (15.67 ± 9.85), HAMD between EM (15.67 ± 9.85) and CM (16.31 ± 10.52), MoCA among NC (26.89 ± 2.47), EM (29.16 ± 1.47), and CM (22.94 ± 5.37). Significant difference was revealed for MIDSA (*P* = 0.000) and onset frequence (*P* = 0.000) between EM and CM (Table 1).

**Table 1:** The clinical characteristics of normal controls, EM patients and CM patients

	NC	EM	CM
Num(M/F)	18 (4/14)	18 (4/14)	16 (4/12)
Age(year)	39.11 ± 9.99	33.39 ± 10.99	42.44 ± 8.65
DD(year)	NA	12.44 ± 8.07	11.25 ± 9.30
VAS	NA	8.33 ± 1.50	7.88 ± 1.45
MIDSA	NA	16.00 ± 17.94	101.81 ± 53.95
Frequence(month)	NA	3.75 ± 2.67	24.81 ± 6.32
HAMA	9.67 ± 3.16	15.67 ± 9.85	21.62 ± 10.98
HAMD	15.89 ± 2.89	10.89 ± 7.26	16.31 ± 10.52
MoCA	26.89 ± 2.47	29.16 ± 1.47	22.94 ± 5.37
Volume	0.32 ± 0.02	0.35 ± 0.02	0.33 ± 0.02

*NC* normal control, *EM* episodic migraine, *CM* chronic migraine, *DD* disease duration, *VAS* visual analogue scale, *MIDSA* migraine disability assessment scale, *HAMA* Hamilton Anxiety Scale, *HAMD* Hamilton Depression Scale, *MoCA* Montreal Cognitive Assessment, *NA* not available

### Comparison of PAG volume among NC, EM and CM groups

**Fig. 2:**
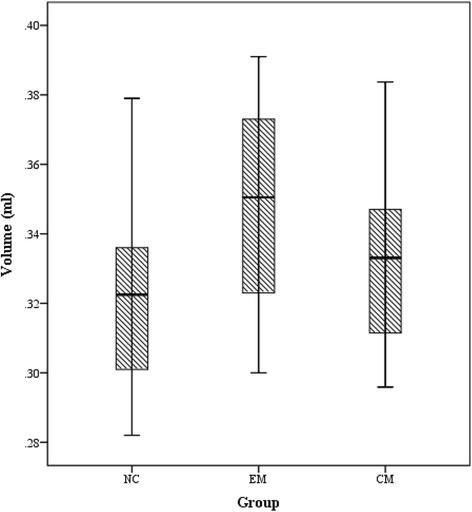
PAG volume of NC, EM and CM patients, whose mean PAG volume is 0.32 ml, 0.35 ml and 0.33 ml, respectively

Table 2 demonstrated that there was a significant difference for PAG volume between NC (0.32 ± 0.02 ml) and EM (0.35 ± 0.02 ml) (*P* = 0.017). Figure 2 indicated that PAG volume of CM (0.33 ± 0.02 ml) fell in between NC and EM, and showed no significance (*P* > 0.05).

**Table 2:** The comparison of PAG volume among groups using one-way analysis of covariance

	Mean difference (95% CI)*	Std. Error.	Sig.^a^
NC vs. EM	−0.023(−0.041 ~ −0.004)	0.009	0.017
NC vs. CM	−0.013(−0.032 ~ 0.006)	0.009	0.170
EM vs. cm	0.01(−0.01 ~ 0.03)	0.010	0.327

^*^The mean difference is significant at the.05 level

^a^Adjustment for multiple comparisons: Least Significant Difference (equivalent to no adjustments), and covariates appearing in the model are evaluated at the following values: Age = 38.15

### Partial correlation analysis between clinical variables and PAG volume

Partial correlation analysis (with age as covariable) showed significant negative correlation of VAS score with PAG volume in CM (*P* = 0.03) (Fig.e 3), and the other clinical variables showed no significant correlation with PAG volume in EM and CM (Table 3).

**Fig. 3:**
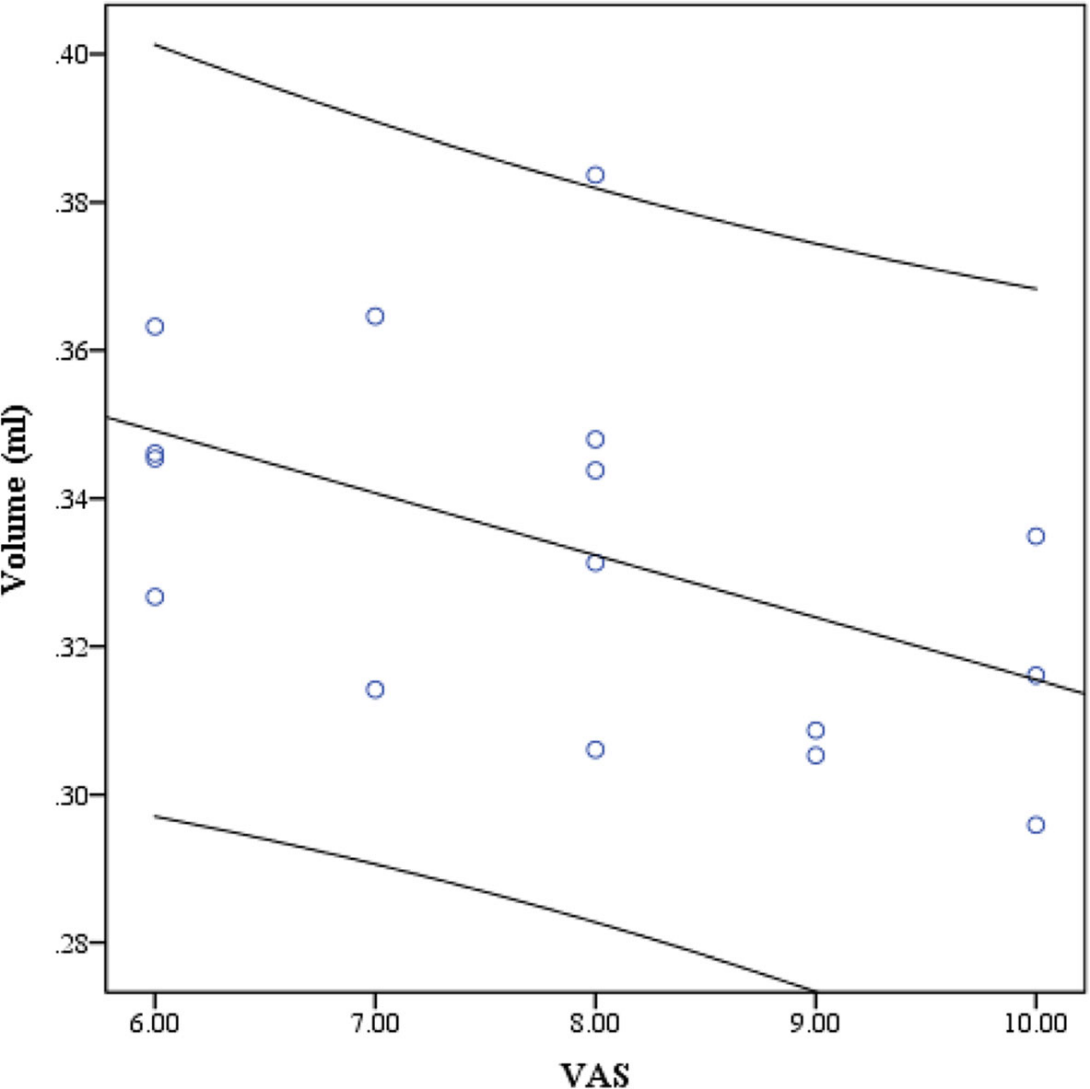
The scatter plot between PAG volume and VAS score in CM, and a negative correlation was revealed (*P* = 0.03)

**Table 3:** The partial correlation analysis between PAG volume and clinical variables

	EM		CM	
	r	*P* value	r	*P* value
DD(year)	0.10	0.36	0.002	0.49
VAS	0.043	0.44	−0.493	0.03
MIDSA	0.094	0.36	−0.291	0.14
Frequence(month)	−0.24	0.17	0.293	0.14
HAMA	0.028	0.46	0.115	0.34
HAMD	0.222	0.20	−0.286	0.15
MoCA	0.058	0.41	−0.025	0.47

### ROC curve analysis among NC, EM and CM groups

**Fig. 4:**
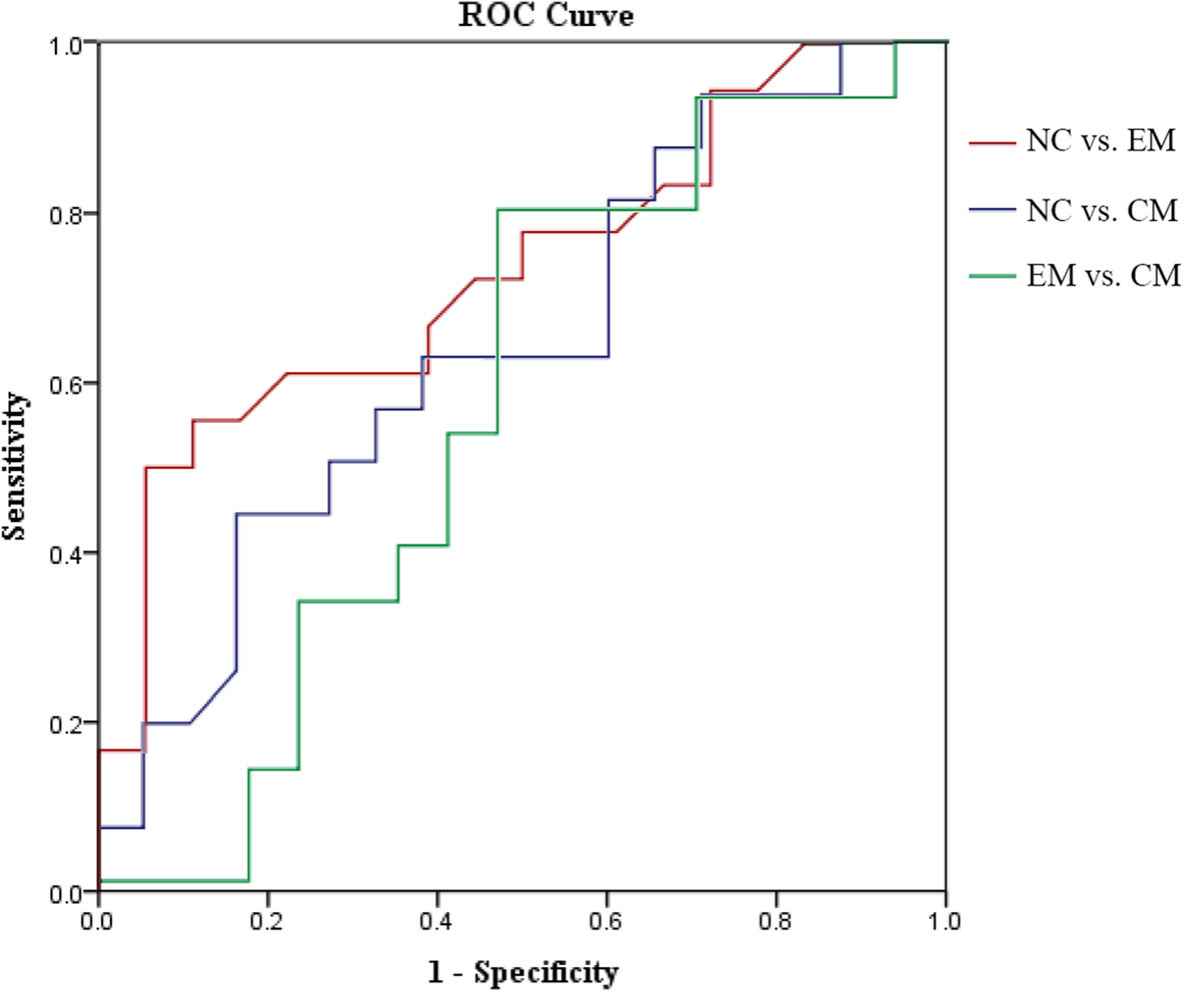
ROC curve among each group, and NC vs. EM had a largest area under the curve (0.731)

Table 4 indicated that PAG volume had a larger AUC in NC vs. EM (0.731) compared with NC vs. CM (0.634) and EM vs. CM (0.618) (Fig. 4). The cut-off value of PAG volume was set as 0.349 ml with sensitivity 0.556 and specificity 0.889 in distinguish EM from NC.

**Table 4:** ROC curve analysis among groups

	Cut-off Value	AUC	Sensitivity	Specificity
NC vs. EM	0.349	0.731	0.556	0.889
NC vs. CM	0.341	0.634	0.438	0.833
EM vs. CM	0.349	0.618	0.813	0.556

## Discussion

In this study, the individual PAG was created by applying the deformation field [22] to the PAG template, and it could be used to compute the true PAG volume. Figure 1 provided a good profile for the PAG template and individual PAG segment, which was completely consistent with the actual PAG location and size.

This study demonstrated that EM had the largest PAG volume, and it was significantly larger than that of NC, which indicated that the PAG volume expansion may take part in the migraine attack. Previous studies demonstrated that PAG lesions may lead to migraine attack [23–25], and functional MRI studies also demonstrated that the PAG network was disrupted in migraine [26, 27]. Therefore, it could be considered that PAG structural change might be the cause of migraine, and PAG volume expansion might be the result of disrupted PAG network in migraine.

Although there was no significant difference on PAG volume within NC-CM and EM-CM groups, the PAG volume of CM showed a slightly reduced tendency compared with EM and slightly increased tendency compared with NC. Therefore, it may speculate that PAG volume reducement may exist in the transformation of EM to CM, and the neuromechanism should be further investigated.

Partial correlation demonstrated that only PAG volume in CM was negatively related to the VAS score, which indicated the PAG volume changes may be associated with VAS score. In EM patients, PAG volume showed no any correlation with the clinical variable, and this point indicated PAG volume expansion may be the direct impairment in EM and may be associated with pathological substrates [13]. The previous study presented that T2-visible load, age, and disease duration may be associated with gray matter volume by VBM methods. Therefore, this study provided a new viewpoint that PAG volume expansion in the migraine patients without T2-visible may be the specific imaging appearance in the midbrain, and it may be an independent brain changes in migraine, which was not infected by T2-load and other clinical factors. Herein, we could speculate that the gray matter changes in migraine may be classified as two patterns: PAG volume expansion and extra-PAG volume reducement based on the current study and previous studies [13, 15, 16].

ROC curve demonstrated that PAG volume expansion may provide a fair level for the diagnosis of EM from NC (AUC = 0.731), and it was not enough to distinguish CM from NC and CM from EM because of lower AUC. Although PAG volume had a fair diagnostic efficacy for EM from NC, and it presented a slightly higher specificity (0.889) and a slightly lower sensitivity (0.556). Based on the Fig. 2, the overlap was observed in between NC and EM, which may decreased the sensitivity for PAG volume as a biomarker. However, it was reasonable to believe that the PAG volume expansion may be inclined to the diagnosis of EM.

Although PAG is a very small structural in the midbrain, this study provided an automated PAG volume measurement methods, and which could be routinely used for the PAG volume measurement in clinical practice. PAG volume expansion could not only be considered as a potential diagnostic imaging biomarker for EM, but also might be considered as a treatment response prognosis for EM just as PAG volume reducement associated with treatment response in medication-overuse headache [16]. The main limit of this study was that the sample of this study was relative small, and it would be necessary to increase the sample size in the future study.

## Conclusion

In conclusion, PAG volume expansion may directly underlie the impairment evidence on the brain in EM, and could be considered as the imaging biomarker for diagnose and evaluation for the migraine.

## Abbreviations

CM: Chronic migraine
EM: Episodic migraine
NC: Normal controls
PAG: Periaqueductal gray
